# Characterization of genital chlamydia amongst female sex workers in Nairobi, Kenya

**DOI:** 10.11604/pamj.2024.47.170.40056

**Published:** 2024-04-08

**Authors:** Priska Bwana, Ferdinard Adungo, Gabriel Magoma, Matilu Mwau

**Affiliations:** 1 Kenya Medical Research Institute, Nairobi, Kenya,; 2 Jomo Kenyatta University of Agriculture and Technology, Nairobi, Kenya

**Keywords:** Female sex workers, *Chlamydia trachomatis*, genital chlamydia infections, genotype, Kenya

## Abstract

**Introduction:**

genital chlamydia, which is caused by diverse Chlamydia trachomatis (C. trachomatis) genotypes, is largely asymptomatic. We aimed to identify C. trachomatis genotypes causing genital chlamydia among female sex workers attending a sex workers outreach program clinic in Nairobi, Kenya.

**Methods:**

this cross-sectional study was conducted between 18^th^ April 2017 and 19^th^ March 2021. Genitourinary complaints from eligible female sex workers were documented using a structured questionnaire. Endocervical swabs were collected for laboratory analysis. C. trachomatis plasmid DNA was extracted, PCR-amplified, and sequenced. Consensus sequences were generated and aligned with reference sequences to determine the C. trachomatis genotypes. Bivariate analysis was used to determine the association between genitourinary complaints and genital chlamydia.

**Results:**

endocervical swabs were collected from a total of 348 participants. Of these, 46 (13.2%) were positive for C. trachomatis. Most (297, 85.3%) of the participants presented with pelvic discharge with or without other symptoms. Fifteen (15, 4.3%) had abdominal pain and 3 (0.9%) had an itchy vulva. There was no statistically significant relationship between clinical presentation and genital chlamydia. Twenty-three samples were successfully sequenced. Each sequence was at least 90% identical to each of the 13 references C. trachomatis genotypes A, B, C, D, E, F, G, Ia, J, L1, L2, L2b and L3.

**Conclusion:**

we found no significant association between individual genitourinary complaints and genital chlamydia infection. The C. trachomatis genotypes circulating amongst female sex workers in Nairobi could be related to genotypes A, B, C, D, E, F, G, Ia, J, L1, L2, L2b, and L3.

## Introduction

Genital chlamydia, caused by *C. trachomatis* [1] is a sexually transmitted bacterial infection [2]. In women, *C. trachomatis* infects the endocervix and causes cervicitis. Since no long-term immunity is induced, re-infection is common among patients. Nineteen different *C. trachomatis* genotypes have been identified, namely A, B, Ba, C, D, Da, E, F, G, Ga, H, I, Ia, J, K, L1, L2, L2a, and L3 [3]. Genotypes A to C are often associated with trachoma, L1 to L3 with lymphogranuloma venereum, and D to K with urogenital and neonatal infections [4]. Genotypes Da, E, F, G, Ga, H, I, Ia, J, and K cause genital chlamydia infections in women [3].

The most common complication of genital chlamydia infection in women is pelvic inflammatory disease (PID). Long-term complications such as fibrosis and scarring due to the repair of tissue damaged by chlamydia-induced inflammation may lead to ectopic pregnancy, infertility, and chronic pelvic pain [5]. In infected pregnant women, the foetus is at risk of conjunctivitis and pneumonia [6]. Although early detection can help prevent these complications, this may be difficult as the infection is often asymptomatic or associated with nonspecific symptoms such as pelvic discharge, genital itchiness, or lower abdominal pain [7]. If untreated, the infection may persist for a long time. Globally, the estimated prevalence of genital chlamydia among female sex workers is between 0.6%-46.2% [8]. Female sex workers are at a high risk of contracting sexually transmitted infections (STIs) [9] and maybe the key drivers of genital chlamydia infections [10]. The risk of contracting genital chlamydia and other STIs is increased by drug and alcohol abuse, inconsistent condom use, multiple sexual partners, mobility, and a complex social network between female sex workers, their clients, and other sexual partners of the clients [11].

In Kenya, studies conducted on genital chlamydia have focused on its epidemiology in targeted populations, and, to the best of our knowledge, there have been no genetic studies. Prevalence rates of 6% [5] have been reported among urban women of reproductive age, 8.8% [12] in pregnant women, 3.2% [13] in fishermen along the Lake Victoria region, and 12% [14] in men who have sex with men. A study conducted among part-time female sex workers in a suburban community in 2002 reported a prevalence rate of 4.2% [9], while another study among Female Sex Workers in an urban community reported a prevalence of 3.1% [15]. Even though, the SWOP City clinic attends to a large number of female sex workers, there have been only limited attempts to describe their socio-demographic characteristics and to associate their genitourinary complaints with the genital Chlamydia they sometimes present. Further, the strains of genital chlamydia circulating among these female sex workers have not been described. In this study, our objectives were to identify *C. trachomatis* genotypes causing genital chlamydia among female sex workers attending a sex workers outreach program (SWOP) City clinic in Nairobi, Kenya, and to determine the association between genitourinary complaints and genital chlamydia.

## Methods

**Study design:** this cross-sectional study was conducted between 18^th^ April 2017 and 19^th^ March 2021. Female sex workers who had genitourinary complaints sought medical services at the sex workers outreach program City clinic in Nairobi, and were able to give informed consent were recruited consecutively to the study.

**Study setting:** the study was conducted in the SWOP City clinic in Nairobi, Kenya. Nairobi, the capital city of Kenya, is an international hub for travel, trade, and economic development. Over the years, Nairobi has experienced rapid population growth and the current population of the city is approximately 4.3 million people. Since it is a trade and travel hub, commercial sex work is a significant economic activity in many parts of the city and the prevalence of STIs such as HIV/AIDS is higher in Nairobi than in the rest of the country. The SWOP City clinic in Nairobi, Kenya is situated in the city´s Central Business District. It has a sexual and reproductive health clinic, and it provides genitourinary medicine services to key populations (KPs) and priority populations in Kenya. Key populations (KPs) include female sex workers (FSW), men who have sex with men (MSM) as well as adolescent girls and young women (AGYW). Recruitment was on a first to consent first to enroll basis until the sample size was achieved. The samples were collected, transported, and tested at the Kenya Medical Research Institute Laboratory for Molecular Biology in Nairobi.

**Study participants:** the study recruited female sex workers who had been advised by the clinician at the health facility to undergo genital Chlamydia testing during their visit for genitourinary medicine services. Pregnant women and patients with serious medical conditions that would have potentially affected the accuracy of normal laboratory tests and the interpretation of results were excluded. The age cut-off for inclusion was 18 years and above, which is the legal age for providing informed consent in Kenya. However, two emancipated minors [16] were enrolled, as the policy on the conduct of HIV and STI research in adolescents allows for the inclusion of emancipated minors since they do not need parental consent.

**Study variables:** this study collected socio-demographic, genitourinary, and laboratory variables. Socio-demographic characteristics included age, level of education, marital status, number of sexual partners in 2 weeks, and history of sexually transmitted infections in the past 1 year. Genitourinary complaints included the presence of color and smell of pelvic discharge, abdominal pain, pain during urination (dysuria), vulva itchiness, and other genitourinary complaints. These variables were collected from the study participants to document the socio-demographic characteristics and the genitourinary complaints the female sex workers at SWOP City clinic present. Endocervical swab samples were collected from the endocervix of the study participants. These were the laboratory test samples for this study and the test outcomes were documented as genital chlamydia positive or negative. The documented genitourinary complaints and genital chlamydia positive test outcomes were used to determine the association between genitourinary complaints and genital chlamydia.

**Sampling and recruitment procedures:** the study team worked closely with clinicians at the SWOP City clinic where the study was conducted. The nurse triaged female sex workers who came to the SWOP city clinic: some of the participants were reviewed by the nurse and given a new appointment date or sent to the pharmacy for medicine. Others were referred to the clinician. Based on the clinical assessment, eligible participants were recruited on a first-to-consent basis. Briefly, after routine consultation, the attending clinician informed the patient of the study, eligibility was assessed and patients who agreed were invited to participate. Written information was obtained from each participant. The rest of the participants were recruited consecutively in the same manner.

**Data sources/measurement:** socio-demographic characteristics and genitourinary complaints were documented using a structured questionnaire administered by the attending clinician. All participants were physically examined, and the attending clinician collected two endocervical swab samples. One swab was used for microscopy while the other swab was sent to the KEMRI laboratory for DNA extraction, PCR amplification, and DNA sequencing. The swabs were kept in cooler boxes at 4°C and couriered to the KEMRI laboratory within three hours of collection.

**Bias:** in this study, selection bias was expected because the study participants did not randomly originate from the community. To minimize bias after enrollment, the persons performing the test were completely blinded to the participant's status and clinical presentation. They were not aware of the patient's status before the samples were collected and the test performed. Some sociodemographic characteristic variables responses were missing for some participants’ data, and this may have introduced reporting bias.

**Sample size:** a sample size of 347 study participants was determined to be sufficient to describe the presence of genital chlamydia with a significance criterion of 0.05. This was calculated using Fischer´s exact test formula [17] as well as prevalence rates from previous studies conducted in Kenya [5]. In the end, 348 participants were recruited.

### Quantitative variables

**Preparation of samples for PCR:** at the laboratory, the samples were assigned unique and sequential numbers and stored at -30°C before DNA extraction. Before testing, the swabs were retrieved, thawed at room temperature, and then cut and put into 1.5ml Eppendorf tubes. Deoxyribonucleic acid (DNA) was extracted from the swabs using the ReliaPrep™ gDNA Tissue Miniprep System (Promega Corporation, 2800 Woods Hollow Road, Madison, Wisconsin, United States) according to the manufacturer´s instructions [18].

**Amplification reaction:** a 241-base pair (bp) fragment of the ORF2 region of the highly conserved *C. trachomatis* cryptic plasmid DNA [19] was amplified to identify *C. trachomatis* using the primer pair KL1 (forward primer) 5'- TCCGGAGCGAGTTACGAAGA-3' and KL2 (reverse primer) 5'-AATCAATGCCCGGGATTGGT-3' (reverse primer) [20]. We chose plasmid primers because the detection of *C. trachomatis* using this method is 10 to 1000 times more sensitive than those based on ribosomal DNA (rDNA) or the major outer membrane protein (MOMP) gene [21,22]. The 50μL PCR reaction mix comprised 25μL HotStar Taq master mix (Qiagen, Hilden Düsseldorf, and Germany), 10μL nuclease-free water, 5μL DNA sample and 10μM each of primers KL1 and KL2. PCR conditions included an initial denaturation step at 95°C for 15 minutes, followed by 35 cycles of denaturation at 93°C for one minute, annealing at 64°C for one minute, and extension at 72°C for one minute. A final extension cycle at 72°C was run for 5 minutes [23]. The amplicons (5μL) were loaded onto a 1.0% agarose gel and electrophoresed at 100v for 50 minutes. The agarose gel was stained in ethidium bromide solution and visualized using a Clearview Ultra Violet Transilluminator (Cleaver Scientific Ltd, Unit 41 Somers Road, Rugby, United Kingdom). Fragment sizes were compared with the 100bp DNA ladder (Qiagen, Hilden Düsseldorf, Germany) to identify *C. trachomatis*; a 241bp fragment size was expected [20].

**DNA sequencing:** polymerase chain reaction (PCR) products were purified with ExoSAP-IT™ (Thermo Fisher Scientific, Waltham, Massachusetts, United States) according to well-established protocols [24]. Forward and reverse sanger sequencing was conducted using BigDye™ Terminator v3.1 Cycle Sequencing kit (Thermo Fisher Scientific, Waltham, Massachusetts, United States). A reaction mix containing a final volume of 11.5μL was prepared from 4μL of nuclease-free water, 2μL of 5x BigDye buffer, 3.5μL of DNA sample, 1μL of Big Dye Terminator v3.1 reaction mix and 4μM of each the KL1 and KL2 primers. The sequencing reaction was run for 25 cycles of denaturation at 96°C for 30 seconds, annealing at 50°C for 5 seconds, and extension at 60°C for 4 minutes [23]. The products were purified using the BigDye XTerminator™ purification kit (Thermo Fisher Scientific, Waltham, Massachusetts, United States) according to the manufacturer's instructions [24]. The purified products were analyzed in the 3730XL DNA analyzer (Thermo Fisher Scientific, Waltham, Massachusetts, United States).

**Statistical methods:** data were transferred from the questionnaires into a Microsoft Excel (Microsoft Corporation, Redmond, Washington, United States) database and analyzed using Stata/MP Version 13 for Windows (StataCorp LLC, College Station, Texas, United States). Quantitative data on socio-demographic characteristics and genitourinary complaints were summarized using descriptive statistics. To facilitate analysis, PCR results were coded as either 1 for positive or 0 for negative and summarized using simple descriptive statistics. Pearson chi-square tests of independence were performed to examine the relationship between participant characteristics, clinical presentation, and genital chlamydia positivity. Consensus DNA sequences were generated using BioEdit software v7.2 (Bioz, Los Altos, California, United States), and *C. trachomatis* genotype identification was done using the Basic Local Alignment Sequence Tool (BLAST) [25] (National Center for Biotechnology Information, Bethesda, Maryland, United States). We used Blastn and Megablast programs as well as the BLAST default parameters to query the nucleotide collection (nr/nt) database.

**Ethical considerations:** the Kenya Medical Research Institute´s Scientific and Ethics Review Unit (SERU) reviewed and approved this study under KEMRI/SERU Protocol No. 3357. The clinicians obtained written informed consent from all the study participants. All but two of the study participants were over 18 years of age. The two participants who were aged below 18 years were considered liberated minors. Written informed consent was obtained from them in line with the existing policy on the conduct of HIV and STI research in adolescents [16]. The endocervical swabs collected from study participants were labeled with unique de-identified study identification numbers to ensure the anonymity of participants.

## Results

**Participants:** three hundred and forty-eight (348) female participants who met the inclusion criteria were enrolled and included in the study.

### Descriptive data

**Participant characteristics:** some participants did not provide complete data on age (10 participants), level of education (6), marital status (2), number of sexual partners in 2 weeks (11), and history of STI (2). Fifty-three-point-eight percent (53.8% (182/338)) of the participants were aged between 15-30 years, while 46.2% (156/338) were aged 31-80 years (Table 1). Seventy-nine point-five percent (79.5% (272/342)) of the participants had obtained secondary school education, 93.6% (324/346) were single, 89.0% (308/346) had not been infected by any STI in the past 1 year, and 39.8% (134/337) reported having had 28 sexual partners or less in the past 2 weeks.

**Table 1:** relationship between genital chlamydia, demographic characteristics and clinical presentation of female sex workers seeking services at the SWOP city clinic, Nairobi, Kenya between 18^th^ April 2017 and 19^th^ March 2021

Participant characteristics	C. trachomatis-negative	C. trachomatis-positive	Degrees of freedom (df)	Chi statistic (X^2^)
Age (years) (n=338*)				
15-30	160 (87.9%)	22 (12.1%)	1	0.3
31-80	134 (85.9%)	22 (14.1%)		
Education (n=342*)				
Primary	57 (81.4%)	13 (18.6%)	1	1.98
Secondary	239 (87.9%)	33 (12.1%)		
Marital status (n=346*)				
Single	283 (87.3%)	41 (12.7%)	1	1.81
Married/cohabiting	17 (77.3%)	5 (22.7%)		
Number of sexual partners in 2 weeks (n=337*)				
0-28	115 (85.8%)	19 (14.2%)	2	0.38
29-56	107 (87.0%)	16 (13.0%)		
57-200	71 (88.8%)	9 (11.3%)		
History of sexually transmitted infections in the past 1 year (n=346*)				
Yes	30 (78.9%)	8 (21.1%)	1	2.23
No	270 (87.7%)	38 (12.3%)		
Pelvic discharge (n=347*)				
Without colour and smell	142 (85.0%)	25 (15.0%)	4	1.08
With colour and smell	9 (90.0%)	1 (10.0%)		
With smell only	60 (87.0%)	9 (13.0%)		
With colour only	45 (88.2%)	6 (11.8%)		
No	45 (90.0%)	5 (10.0%)		
Pain (abdominal pain and dysuria) (n=347*)				
Yes	15 (100%)	0 (0.0%)	1	2.39
No	286 (86.1%)	46 (13.9%)		
Itchy vulva with pain and pelvic discharge (n=347*)				
Yes	39 (84.8%)	7 (15.2%)	1	0.18
No	262 (87.0%)	39 (13.0 %)		
Other genitourinary complaints (n=347*)				
Yes	3 (100%)	0 (0.0%)	1	0.46
No	298 (86.6%)	46 (13.4%)		

* Participants did not provide complete data for this variable

**Genitourinary complaints:** a total of 330 (94.8 %) of the 348 participants had at least one genitourinary complaint, while 18 (5.17%) had none. Two hundred and ninety-seven (297/347 85.6%) participants presented with pelvic discharge with or without other symptoms. Fifteen (15, 4.3%) participants complained of abdominal pain, nine had lower abdominal pain, while six had pain during urination. Forty-six (46/347, 13.3%) participants had an itchy vulva as the main complaint, 43 had an itchy vulva accompanied by pain and pelvic discharge, while 3 had an itchy vulva only. Three (3/347, 0.9%) participants presented with bleeding from the cervix, genital wounds, and rash in the mons pubis.

**Figure 1:**
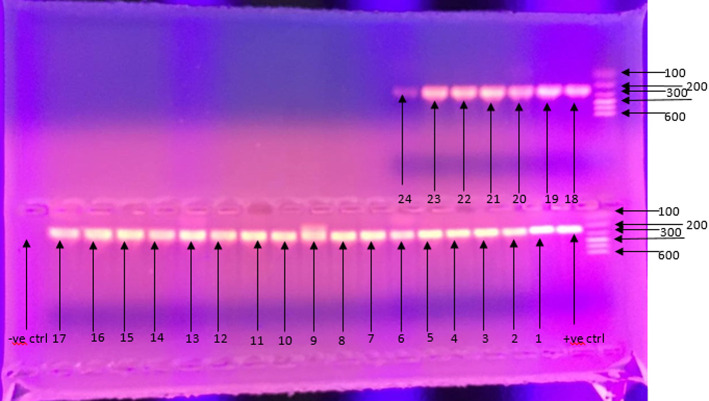
agarose gel electrophoresis (1% agarose) of PCR products for *C. trachomatis* positive samples; lower agarose gel wells from the right: DNA size marker of 100bp in well 1, positive control in well 2, *C. trachomatis* positive sample numbers 1 to 17 in wells 3-19 and negative control in well 20; upper agarose gel wells from the right: DNA size marker of 100bp in well 1 and *C. trachomatis* positive sample numbers 18-24 in wells 2-8

**Figure 2:**
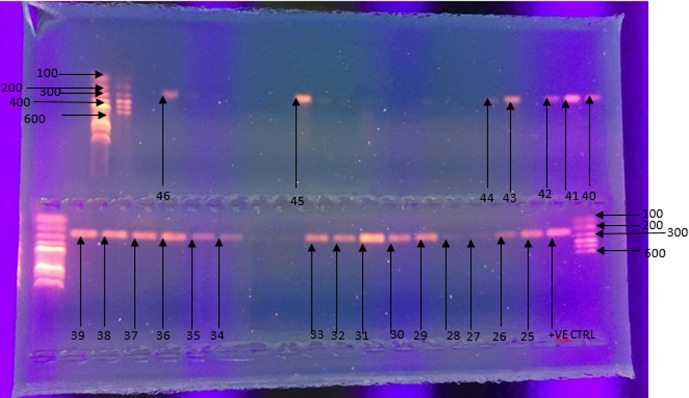
agarose gel electrophoresis (1% agarose) of PCR products for *C. trachomatis* positive samples; the lower agarose gel wells from the right: DNA size marker of 100bp in well 1, positive control in well 2, *C. trachomatis* positive samples number 25-39 in wells 2-11 and 14-19; the upper agarose gel wells from the right: *C. trachomatis* positive samples number 40-46 in wells 1-3, 5 and 6, 15, and well 21; the DNA size marker of 100bp is in well 23

**Outcome data:** of the 348 samples tested by PCR, 46/348 (13.2%) were positive for *C. trachomatis*, while 302/348 (86.8%) were negative. The agarose gel electrophoresis images for the samples that gave positive outcomes are provided in Figure 1 and Figure 2.

**Association between genitourinary complaints and genital chlamydia:** in all cases, there was no significant relationship between genitourinary complaints and genital chlamydia. Only one of the 18 participants who had no symptoms tested positive for *C. trachomatis*, while three (3, 25.0%) of the 12 participants who provided no information or complaints were positive. Similarly, of the 297 participants who presented with pelvic discharge with or without other symptoms, 41 (13.8%) tested positive for *C. trachomatis*. One (1, 33.3%) of the three participants with an itchy vulva without other symptoms tested positive for *C. trachomatis*. None of the 15 participants presented with either lower abdominal pain or pain during urination, as their only complaint were positive for *C. trachomatis*. Also, none of the three (3, 100%) participants who had bleeding from the cervix, genital wounds, and rash in the mons pubis were positive for *C. trachomatis*.

***Chlamydia trachomatis* genotypes:** of the 46 samples that tested positive for *C. trachomatis*, 40 had sufficient PCR product for subsequent sequencing. Twenty-three of these were successfully sequenced, while 17 failed quality checks due to suboptimal DNA quality. The 23 DNA sequences were deposited in GenBank (National Centre for Biotechnology Information, Bethesda, Maryland, United States). Their accession numbers are provided in Table 2. Each of the 23 sequences was at least 90% identical to each of the 13 reference *C. trachomatis* genotypes A, B, C, D, E, F, G, Ia, J, L1, L2, L2b, and L3 (Annex 1).

**Table 2:** accession numbers of sequences obtained from commercial sex workers in an outreach clinic in Nairobi, Kenya, between 18^th^ April 2017 and 19^th^ March 2021

Study sample sequence	Accession number
SWOP 03	OM398457
SWOP 10	OM398458
SWOP 11	OM398459
SWOP 87	OM398460
SWOP 142	OM398461
SWOP 162	OM398462
SWOP 164	OM398463
SWOP 170	OM398464
SWOP 185	OM398465
SWOP 185B	OM398466
SWOP 207	OM398467
SWOP 210	OM398468
SWOP 224	OM398469
SWOP 246	OM398470
SWOP 381	OM398471
SWOP 394	OM398472
SWOP 433	OM398473
SWOP 461	OM398474
SWOP 492	OM398475
SWOP 499	OM398476
SWOP 519	OM398477
SWOP 569	OM398478
SWOP 624	OM398479

## Discussion

This study reports a genital chlamydia prevalence of 13.2% among female sex workers attending the SWOP City clinic in Nairobi. This differs from the previously reported rates of 3.1% [15] and 9% [26] in Kenya and 6.8% [27] in sub-Saharan Africa among female sex workers in the community. The observed higher prevalence rate in the current study could be attributed to the recruitment strategy. The female sex workers in this study were all recruited from a sex workers clinic. Furthermore, the risk factors for genital chlamydia could be different in each of these studies and the dynamics of the sexual networks that sustain STIs may differ between these study groups [28]. For instance, super-spreader events that are well described in other infectious diseases like the severe acute respiratory syndrome (SARS) pandemic [29] may also affect the dynamics of genital chlamydia transmission within a community. Nonetheless, our findings fall within the global estimated genital chlamydia prevalence of between 0.6%-46.2% among female sex workers [8]. Local populations of female sex workers may have varying periods of high and low genital chlamydia prevalence based on extant population dynamics.

Globally, sexually transmitted infections are managed symptomatically. However, genital chlamydia tends to be largely asymptomatic [30-32]. Interestingly, in our study, 42 (91.30%) of the 46 participants who had genital chlamydia had genitourinary signs and symptoms such as pelvic discharge, itchy vulva, and lower abdominal pain/dysuria. These signs and symptoms appear not to be pathognomonic. It should be noted that the proportion of genital chlamydia-positive patients with signs and symptoms may not reflect the true population rate, as female sex workers who had no symptoms but may have had genital chlamydia had no reason to visit the SWOP City clinic. The current practice is to treat patients presumptively without laboratory testing. Even though this is highly effective, we propose that a simple and accurate multiplex laboratory test that can be applied at the point of care could help identify multiple pathogens that cause genitourinary infections [33].

Different *C. trachomatis* genotypes are associated with different genitourinary signs and symptoms. We sequenced a small number of samples. However, sequences of the short fragment of cryptic plasmid we sequenced did not yield sufficient resolution to distinguish different *C. trachomatis* genotypes. As such, we are not able to draw a distinct association between the *C. trachomatis* phenotypes and genotypes. Regardless of the genotype responsible, genital tract infections may cause a broad spectrum of clinical symptoms [34]. This is not surprising, as other studies have found that multiple genotypes may account for diverse clinical outcomes [7,35-37].

**Study limitations:** we have not tested for other STIs yet, STIs usually occur as co-infections. Therefore, testing for a broad range of pathogens could help explain many of the signs and symptoms we observed. Also, data on genital chlamydia is scanty, and it is therefore difficult to fully compare our findings with others. Lastly, the ∼241bp sequences obtained in this study did not provide sufficient resolution to identify distinct genotypes. In the future, we can address this limitation by sequencing large fragments of the *ompA* gene.

## Conclusion

We found no significant association between individual genitourinary complaints and genital chlamydia infection. The *C. trachomatis* genotypes that are circulating amongst female sex workers in Nairobi could be related to genotypes A, B, C, D, E, F, G, Ia, J, L1, L2, L2b, and L3 but not genotypes Ba, Da, Ga, H, I, K and L2a.

### What is known about this topic

- *Female sex workers are at high risk of genital chlamydia infection;*
- *Genital chlamydia infection can be symptomatic and asymptomatic;*
- *There are Chlamydia trachomatis genotypes circulating in Nairobi.*

### What this study adds

- *Thirteen percent of the female sex workers under the study tested positive for Chlamydia trachomatis;*
- *There was no significant relationship between genitourinary complaints and testing positive for Chlamydia trachomatis;*
- *The Chlamydia trachomatis genotypes that are circulating amongst female sex workers in Nairobi could be related to genotypes A, B, C, D, E, F, G, Ia, J, L1, L2, L2b and L3.*

## Annex

**Annex 1**: BLAST results of sequences obtained from commercial sex workers in an outreach clinic in Nairobi, Kenya, between 18^th^ April 2017 and 19^th^ March 2021 (PDF - 209 Kb)
